# Triage after drug overdose: effect of the Introduction of a medical psychiatry unit on the allocation of ICU beds

**DOI:** 10.1186/cc14593

**Published:** 2015-03-16

**Authors:** D Kleinloog, F Braam Houckgeest, D Sierink

**Affiliations:** 1 Tergooiziekenhuizen, Hilversum, the Netherlands

## Introduction

Many patients with drug overdose are sedated, but do not have medical reasons to warrant ICU admission. Historically, monitoring behavior and suicide risk was done in the ICU, until the patient was awake enough for psychiatric consultation.

## Methods

A medical psychiatry unit (MPU) was instituted as part of the Department of Clinical Psychiatry. For all patients with drug overdose in the emergency department, a risk assessment was made by the intensivist. Those without ICU indication (such as cardiac or respiratory monitoring) were admitted to the MPU. Alternatively, when awake enough, they were seen by the psychiatrist immediately. We performed an analysis of all patients with drug overdose, admitted to our ICU (before MPU *n *= 88, after MPU *n *= 191). We used the Welch *t *test for comparisons.

## Results

After institution of the MPU, there was a 28% reduction in the number of patients with drug overdose per month, admitted to the ICU. Also, patients admitted to the ICU were sicker and stayed longer (see Table 1). There were no patients admitted to the ICU after initial MPU admission.

**Table 1:** Patient numbers and disease severity before and after Introduction of MPU.

	Before MPU (18 months)	Since MPU (51 months)	Difference	95% CI	*P *value
Admissions per month	4.9	3.5	-1.4	-2.7 to -0.1	0.04
APACHE II score	8.4	12.5	+4.0	+1.8 to +6.2	0.0004
APACHE III score	30.9	40.3	+ 9.4	+2.6 to +16.2	0.0069
Length of stay	0.8	1.3	+ 0.5	-0.0 to +1.0	0.05

## Conclusion

Introduction of an MPU was associated with reduced numbers of patients with drug overdose admitted to the ICU. Those admitted to the ICU after the institution of the MPU were sicker, probably indicating more appropriate use of ICU beds.

